# Concerning the Obstetrical Properties of Ergot of Rye

**Published:** 1869-05

**Authors:** 


### Concerning the Obstetrical Properties of Ergot of Rye.

M. Ameville presented the following case, which gave rise to a discussion upon the properties of the ergot of rye, in the Société Medico-Pratique de Paris:

On the 24th of last May, I was called by a midwife to see a lady, thirty years of age, large, strong, and a primipara. Two hours after the child was delivered, the placenta not having come away, the midwife had administered some ergot of rye; but instead of producing thereby the expulsive pains she had expected, the uterine contractions confined themselves to the muscular fibres of the neck, which closed completely. When I arrived the child had been delivered about five hours. The os would admit only with difficulty the end of the finger; the introduction of the hand to reach the placenta was not to be thought of. Both the midwife and family were greatly alarmed, because from time to time there were slight discharges of blood; and the midwife, fearing a hemorrhage, did not dare leave the patient. Having in the first place reassured their minds, I ordered that an injection of tepid water be made upon the neck of the uterus for eight or ten minutes, and that this be repeated if necessary at the end of an hour. I returned two hours after and found that the spasm of the neck had almost entirely yielded, and that the os was supple and sufficiently dilated to admit the end of the hand shaped into a cone. I therefore gradually produced complete dilatation, and having introduced the hand and detached the placenta, which was still adherent at one of its edges, I completed the labor.

I cite this case, to demonstrate to you once more the impropriety of administering ergot of rye in certain circumstances, in which, on the contrary, direct intervention should be resorted to; and also how its administration may hinder, at least momentarily, the performance of the necessary procedure; and again, to show the influence of tepid injections upon dilatation of the os.—*L'Union Médicale, No.* 24, 1869.

